# Development and validation of a high throughput whole blood thrombolysis plate assay

**DOI:** 10.1038/s41598-017-02498-2

**Published:** 2017-05-24

**Authors:** T. Bonnard, L. S. Law, Z. Tennant, C. E. Hagemeyer

**Affiliations:** 1 NanoBiotechnology Laboratory, Australian Centre for Blood Diseases, Monash University, Melbourne, Australia; 2 VascularBiotechnology Laboratory, Baker Heart and Diabetes Institute, Melbourne, Australia; 3 RMIT University, Melbourne, Australia

## Abstract

The objective of this work was to develop a high throughput assay for testing *in vitro* the thrombolytic activity using citrated whole blood samples, and to overcome the limitations of currently available techniques. We successfully developed a method that involves forming halo shaped, tissue factor induced, whole blood clots in 96 well plates, and then precisely measuring the thrombolysis process with a spectrophotometer plate reader. We here describe the implementation of this novel method, which we refer to as halo assay, and its validation with plasmin, urokinase and tissue plasminogen activator at different doses. The resulting data is a highly detailed thrombolysis profile, allowing comparison of different fibrinolytic agents. The time point analysis allows kinetic data to be collected and calculated to determine key parameters such as the activation time and the rate of fibrinolysis. We also assessed the capacity of the model to study the effect of clot maturation time on the fibrinolytic rate, an aspect of thrombosis rather unexplored with currently available methods, but of increasing importance in drug development. This novel thrombolysis assay could be an extremely useful research tool; to study the complex process of thrombolysis, and a valuable translational clinical tool; as a screening device to rapidly identify hypo- or hyper-fibrinolysis.

## Introduction

Pharmacological fibrinolytic therapy is currently the most prevalent treatment for thromboembolic diseases such as acute stroke or myocardial infarction^1, 2^. Blood clot dissolution is obtained by the lysis of fibrin meshwork via the action of plasmin (or a truncated form of plasmin) injected locally via a catheter, or generated via plasminogen activators administered systemically^3–5^. Unfortunately, these drugs are associated with life-threatening bleeding complications, along with neurotoxic side effects, and the average benefit to risk ratio provided is too low^6^. Acute thrombosis treatment could be improved by the implementation of new techniques which would provide rapid assessment of the haemostasis state of patients on thrombolytic therapy. Additionally the research and development of novel thrombolytic agents requires simple, precise and reproducible *in vitro* methods to investigate mode of action and determine activity.

The traditional method of thrombolysis measurement involves direct manual handling of the aggregates; introducing errors and limiting precision^7^. Rheology techniques based on the viscoelastic properties of blood clot formation and dissolution (i.e. thromboelastography, rotational thromboelastometry or free oscillation rheometry) are suitable to determine the fibrinolytic rate, but are limited by equipment availability, required sample size (300 μL of whole blood) and the number of tests which can be run at a time (4 samples maximum)^8–10^.

A current alternative method, often referred to as “euglobulin clot lysis time” (ECLT) or “overall haemostatic potential”, involves measuring the lysis of plasma aggregates via the decrease in turbidity (absorbance at 405 nm)^11, 12^. This approach has the advantage of being translatable to a microtiter plate, and therefore enables high throughput screening with a basic microplate spectrophotometer^13, 14^. However, this type of test is exclusively performed with plasma samples, cutting out all the circulating cells which play a major role in thrombolysis; (i) platelets, which release fibrinolysis inhibitors and whose aggregation and contraction increases the stiffness of the thrombus^15, 16^, (ii) leukocytes, which form mixed aggregates with platelets, thus affecting thrombus structure, and may also have an anti-fibrinolytic effect via secreting proteases (which enhance platelet adhesion and form neutrophil extracellular traps^17, 18^), and (iii) considering some reports suggest that the haematocrit could also influence the rate of thrombolysis^19, 20^.

Although clot maturation and contraction are not yet fully understood, this phenomenon is commonly associated with thrombolysis resistance, due to a combination of increased stiffness (which limits solute transport), and an accumulation of anti-fibrinolytic molecules^21–24^. However, this has been poorly confirmed experimentally. The setting of the viscoelastic assay implies that the fibrinolytic agent is added at the beginning of the assay and thus does not allow study of the consequences of clot maturation. Sutter *et al*. verified on whole blood aggregates formed within Pasteur pipettes that the thrombolysis from recombinant tissue plasminogen activator (rt-PA) is less efficient on retracted stiff clots than on unretracted softer clots^25^. Additionally Holland *et al*. showed with a similar model that rt-PA degrades the freshly formed clots (3 hours) to a greater extent than clots aged for more than one day^26^. These studies confirm that thrombus ageing and contraction are important factors which impair thrombolysis.

**Figure 1:**
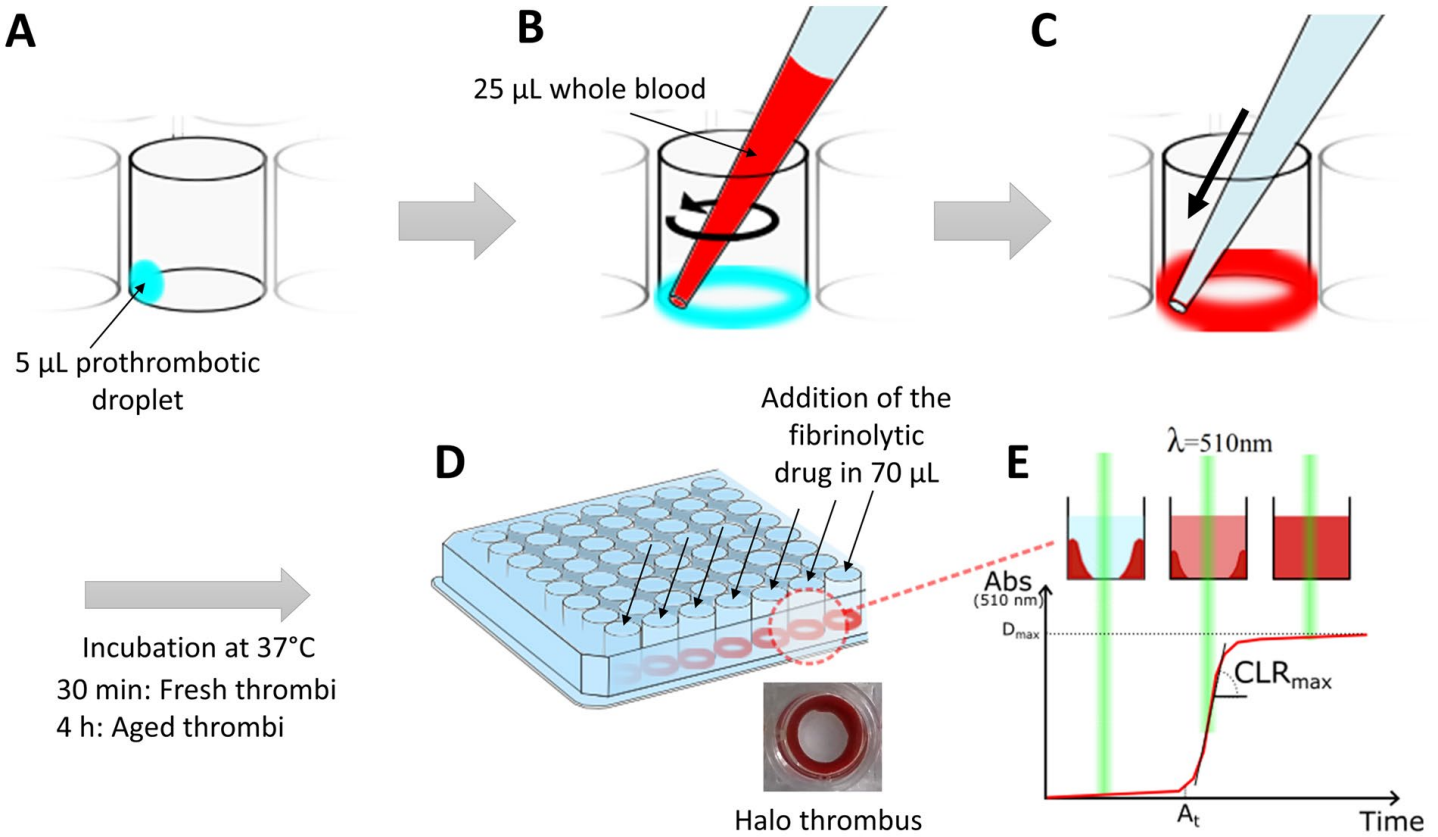
Schematic illustration of the halo assay protocol. (**A**) Droplets of the clotting mixture (Innovin + CaCl_2_) are deposited on the bottom edge of the wells of a 96 well plate. (**B**) The clotting mixture is spread around the edge of the wells with the tip of a P100 micropipette containing 25 μL of blood. (**C**) The blood is slowly released and mixed around the edge of the well thereby making use of the fluidic cohesion effect. (**D**) After incubation at 37 °C for 30 minutes (fresh clots) or 4 hours (aged clots), the clots should have a homogenous halo shape at the bottom of the wells, leaving the centre area of the well clear and empty. (**E**) The fibrinolytic drugs to be tested are added with a multichannel pipette and the degradation of the halo clots measured straight after with a plate reader at 510 nm from the absorbance of the blood starting to progressively cover the centre of the well. Several parameters are determined on the obtained fibrinolysis profiles; the maximum degradation (D_max_), the activation time (A_t_) and the maximum clot lysis rate (CLR_max_).

In this study, we describe an easy to implement, high throughput, whole blood based *in vitro* thrombolytic assay which might overcome the limitations of current methods. The method consists of monitoring the lysis of halo shaped clots via the change in absorbance due to red blood cells released by degradation, as measured by plate spectrophotometry (overview in Fig. 1). The technique enables a drastic reduction of blood volume (25 µL compared to 300 µL for TEG or ROTEM), while keeping the measurements relevant to thrombosis physiopathology, as it does not exclude a large amount of blood components (compare to ECLT assay which is restricted to the euglobulin fraction). The implementation of this technique also offers the possibility to investigate the impact of clot maturation on thrombolysis.

## Results

### Fibrinolysis profiles with plasmin, urokinase and tissue plasminogen activator

The method to monitor, via spectrophotometry, the degradation of the halo clots incubated for 30 minutes at 37 °C was assessed with the addition of defined concentrations of human plasmin, urokinase and recombinant tissue plasminogen activator (t-PA). The assay provided robust repeatability of thrombolysis monitoring on fresh clots, with an intra-individual coefficient of variation of 5.15%. Importantly the thrombolysis profile from different donors showed variability, which was evidenced by an inter-individual coefficient of variation of 23.59%.

**Figure 2:**
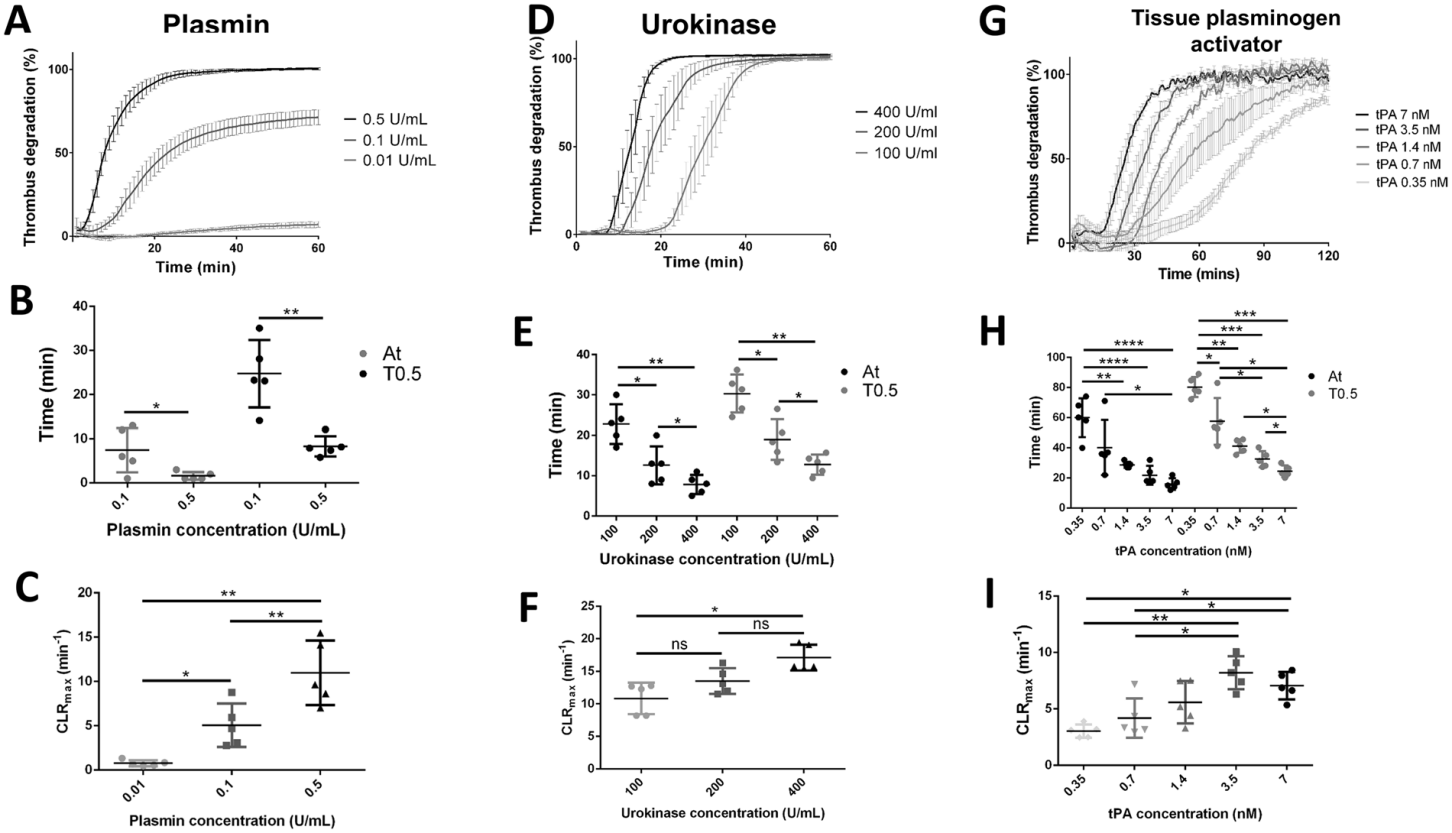
Thrombolysis study with plasmin, urokinase and tissue plasminogen activator (t-PA). Thrombolysis profiles measured with the Halo assay method with 0.5 U/mL, 0.1 U/mL and 0.01 U/mL plasmin (**A**), with 400 U/mL, 200 U/mL and 100 U/mL urokinase (**D**) and with 0.35 nM, 0.7 nM, 1.4 nM, 3.5 nM and 7 nM t-PA (**G**). Activation times (At, black spots) and time to 50% lysis (T0.5, grey spots) (**B**,**E** and **H**) and maximal clot lysis rates (**C**,**F** and **I**) were presented for each concentration as mean values ± SD (n = 5, ns: not significant, *p < 0.05, **p < 0.01, ***p < 0.001, ****p < 0.0001).

The addition of plasmin resulted in a thrombolysis profile that was dependent on the dose tested (Fig. 2A). At the high dose of 0.5 U/mL, a full degradation (over 95%) was obtained after 22 ± 8 minutes, with a short time to 50% lysis, whereas at 0.1 U/mL, the degradation was limited to 70 ± 10% degradation within the first 60 minutes and a longer time to 50% lysis (Fig. 2B, T0.5 = 2 ± 7 min for 0.1 U/mL Vs T0.5 = 8 ± 2 min for 0.5 U/mL, *p < 0.05). At 0.1 U/mL, a short activation time (defined as the time period before initiation of degradation) of 7 ± 4 min was measured, and at 0.5 U/mL the activation time was close to 0 (2 ± 1 min), indicating immediate initiation of thrombolysis. At 0.01 U/mL almost no degradation was observed and no time to 50% lysis or activation time could be measured. Conversely, increasing doses of plasmin resulted in higher maximum clot lysis rates; the 0.5 U/mL samples yielded a degradation rate over twice as fast as the 0.1 U/mL samples (Fig. 2C, 11 ± 3 min^−1^ Vs 5 ± 2 min^−1^).

The addition of plasminogen activators (urokinase and t-PA) resulted in a different degradation profile (Fig. 2D and G). The activation time decreased with increasing concentration of urokinase, as shown in Fig. 2E; 23 ± 4 min with 100 U/mL, 13 ± 4 min with 200 U/mL and 8 ± 2 min with 400 U/mL. The maximum clot lysis rate, presented in Fig. 2F, slightly increased with the concentration; 11 ± 2 min^−1^ for 100 U/mL, 14 ± 2 min^−1^ for 200 U/mL and 17 ± 2 min^−1^ for 400 U/mL. However, all urokinase concentrations tested resulted in a full degradation of the halo clots. The t-PA was tested at lower doses ranging between 0.35 nM and 7 nM. The degradation profiles were also overall dose dependant (Fig. 2G). The activation time and the time to 50% lysis were prolonged at low doses, over 1 hour at 0.35 nM t-PA (Fig. 2H, At = 60 ± 12 min and T0.5 = 80 ± 6 min). At the higher doses, although the activation times were similar to the low urokinase concentrations tested, the maximal clot lysis seemed to be limited to a slower rate (Fig. 2I); at the highest t-PA dose of 7 nM, a CLR_max_ of 7 ± 2 min^−1^ with an activation time of 16 ± 3 min were measured, whereas 100 U/mL urokinase exhibited a faster lysis (11 ± 2 min^−1^) with a slower activation (23 ± 4 min).

### Effect of thrombus maturation on thrombolysis

**Figure 3:**
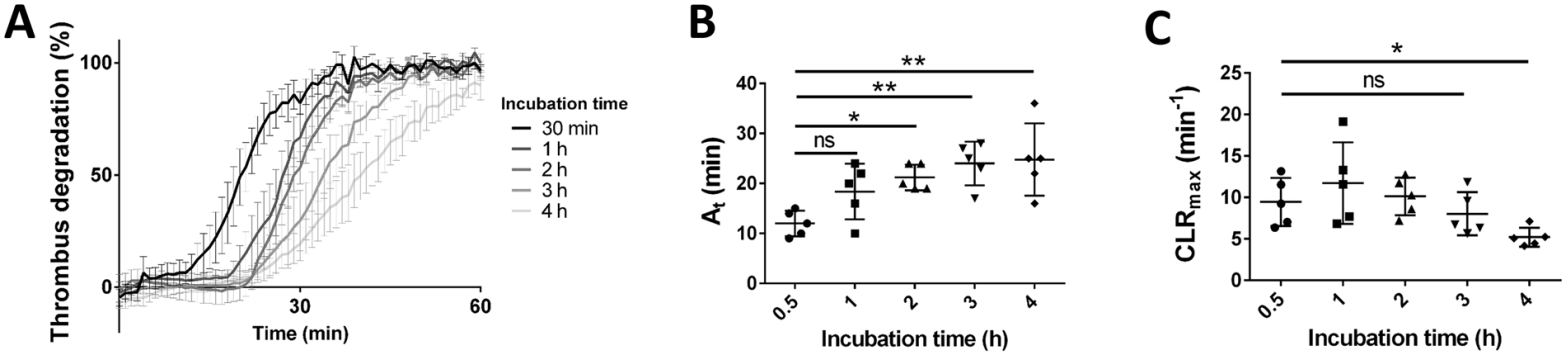
Effect of incubation time. (**A**) Thrombolysis profiles with 200 U/mL urokinase were measured with the Halo assay method on thrombi incubated for 30 min, 1 h, 2 h, 3 h and 4 h. Activation times (**B**) and maximal clot lysis rates (**C**) were presented for each incubation time as mean values ± SD (n = 5, ns: not significant, *p < 0.05, **p < 0.01).

To study the effect of clot maturation in this model, the thrombolysis of halo clots preincubated for 30 minutes, 1, 2, 3 or 4 hours was determined with the addition of 200 U/mL urokinase (Fig. 3). The thrombolysis profiles revealed that the thrombolysis is progressively delayed along with increased preincubation time (Fig. 3A). The activation time seems to increase within the first hours of incubation, and become significantly different from 2 hours incubation (Fig. 3B, 21 ± 2 min Vs 12 ± 2 min on halo clots incubated 30 minutes). The maximum clot lysis rate appeared to require longer incubation to be affected, and was significantly lower only from 4 hours incubation (Fig. 3C, 5 ± 1 min^−1^ Vs 9 ± 3 min^−1^ on halo clots incubated 30 minutes).

**Figure 4:**
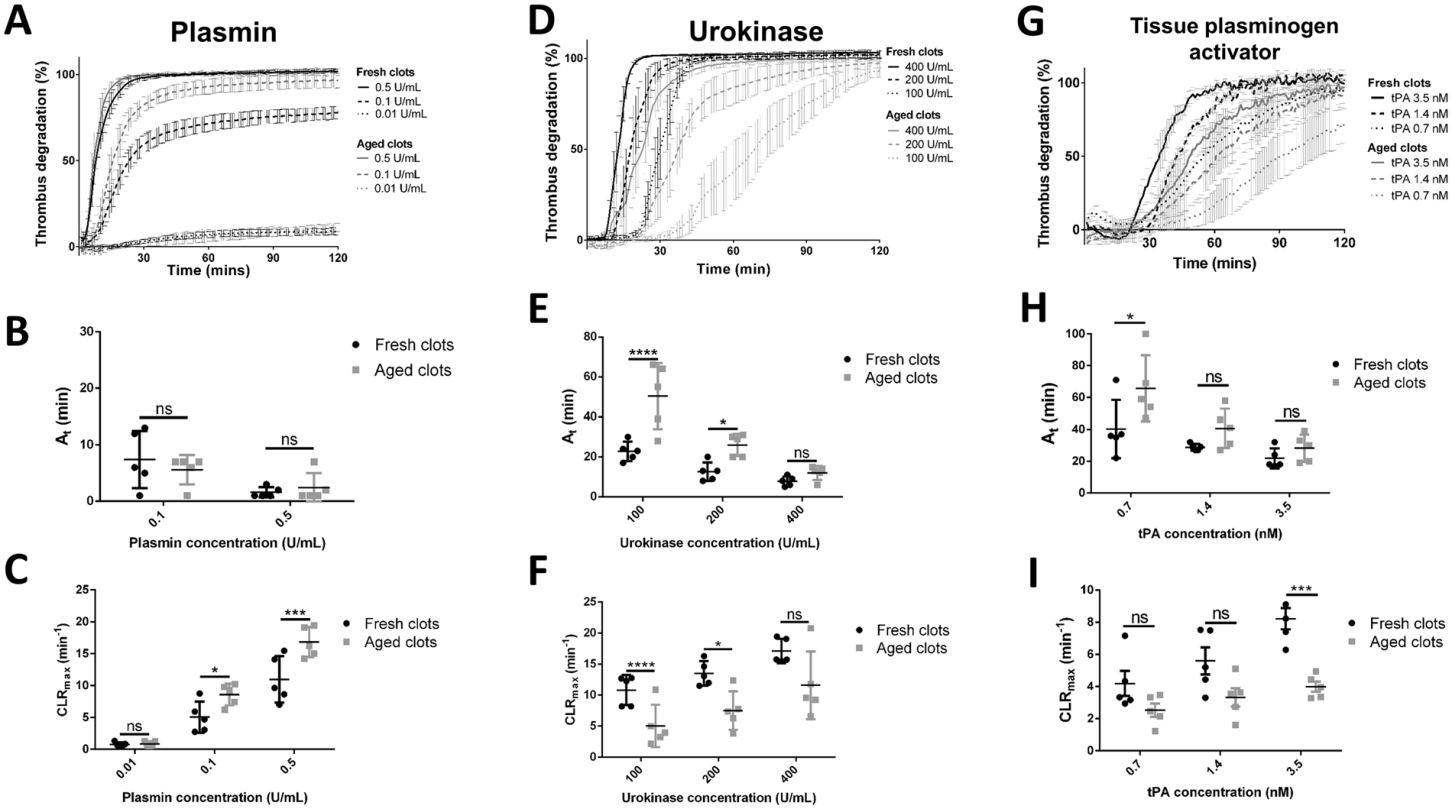
Effect of thrombus maturation on thrombolysis. Thrombolysis profiles were measured with the Halo assay method after 30 minutes incubation (Fresh clots) and after 4 hours incubation (Aged clots) with 0.5 U/mL, 0.1 U/mL and 0.01 U/mL plasmin (**A**), with 400 U/mL, 200 U/mL and 100 U/mL urokinase (**C**) and with 0.35 nM, 0.7 nM, 1.4 nM, 3.5 nM and 7 nM t-PA (**F**). Activation times (**D** and **G**) and maximal clot lysis rates (**B**,**E** and **H**) were presented for each concentration as mean values ± SD (n = 5, ns: not significant, *p < 0.05, **p < 0.01, ***p < 0.001, ****p < 0.0001).

The effect of clot maturation on the thrombolysis was investigated with various concentrations of human plasmin, urokinase and t-PA, and halo clots incubated for 4 hours (aged clots) and 30 minutes (fresh clots) were compared (Fig. 4). The repeatability of the method on aged clots appeared to be less strong than on fresh clots, with an intraindividual coefficient of variation of 9.79%. The thrombolysis rate from different donors showed an important variability, with an interindividual coefficient of variation of 35.48%.

The maturation of the clot seemed to only slightly effect the thrombolysis by plasmin (Fig. 4A). The time to reach full degradation with 0.5 U/mL plasmin changed from 22 ± 7 min (fresh clots) to 16 ± 5 min (aged clots) but was not significantly different (p = 0.2004). The activation time was not significantly affected by clot maturation at 0.1 U/mL or 0.5 U/mL plasmin (Fig. 4B). The maximum degradation obtained with 0.1 U/mL over 2 hours was restricted to 80 ± 7% degradation on fresh clots, and reached 95 ± 6% on aged clots (*p < 0.05). The low concentration of 0.01 U/mL did not induce any apparent degradation. The maximum thrombolysis rate was higher on aged clots with both 0.1 U/mL and 0.5 U/mL plasmin (Fig. 4C, at 0.1 U/ U/mL CLR_max_ mean values of 5 ± 2 min^−1^ on fresh clots Vs 9 ± 2 min^−1^ on aged clots, and at 0.5 U/mL CLR_max_ mean values of 11 ± 3 min^−1^ on fresh clots Vs 17 ± 2 min^−1^ on aged clots).

On the other hand, lysis with urokinase and t-PA was overall less efficient on aged clots (Fig. 4D and G). For 100 U/mL and 200 U/mL urokinase, the times required to initiate the degradation of clots were significantly prolonged by maturation (Fig. 4E). For 100 U/mL urokinase, the activation time was doubled; 23 ± 4 min on fresh clots versus 50 ± 15 min on aged clots. At 200 U/mL, it increased from 13 ± 4 min on fresh clots to 26 ± 5 min. The maximum clot lysis rates were significantly slowed down at those same doses (Fig. 4F). At 100 U/mL the maximum clot lysis rate reduced from 11 ± 2 min^−1^ on fresh thrombi to 5 ± 3 min^−1^ on aged clots, and at 200 U/mL from 14 ± 2 min^−1^ to 8 ± 3 min^−1^. For lysis by t-PA, the effect of thrombus maturation was less prominent. The activation time and the maximum clot lysis rate were significantly different for only 1 out of 3 doses tested; at 0.7 nM t-PA, the At changed from 40 ± 16 min on fresh clots to 66 ± 19 min on aged clots and at 3.5 nM, the CLR_max_ reduced from 8 ± 1 min^−1^ to 4 ± 1 min^−1^.

### Inhibition of GPIIb/IIIa reduces the thrombolysis resistance conferred by maturation

**Figure 5:**
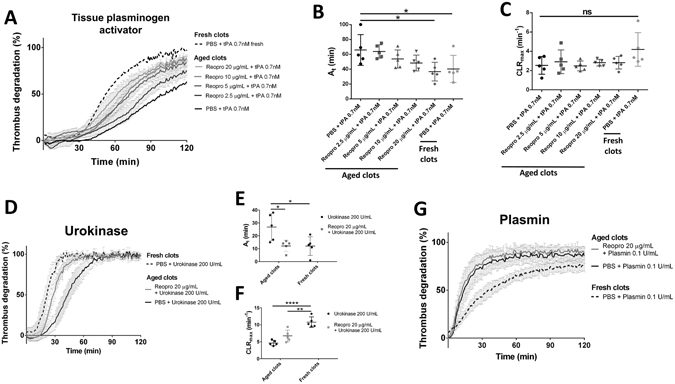
GPIIb/IIIa blockage reduces the thrombolysis resistance conferred by incubation time. Thrombolysis profiles with 0.7 nM tPA were measured on halo thrombi incubated for 4 hours (aged clots) with different concentration of GPIIb/IIIa blocker Reopro (0, 2.5, 5, 10 and 20 µg/mL) and compared to thrombolysis obtained with 0.7 nM tPA on fresh clots (**A**). Activation times (**B**) and maximal clot lysis rates (**C**) were presented for each group as mean values ± SD (n = 5). Thrombolysis profiles with 20 U/mL urokinase (**D**) and with 0.1 U/mL plasmin (**G**) on halo thrombi incubated for 4 hours (aged clots) with or without 20 µg/mL GPIIb/IIIa blocker Reopro and compared to thrombolysis obtained with 20 U/mL urokinase or 0.1 U/mL plasmin respectively. Activation times (**E**) and maximal clot lysis rates (**F**) were presented for the urokinase groups as mean values ± SD (n = 5). ns: not significant, *p < 0.05, **p < 0.01, ****p < 0.0001).

The addition of Reopro at 2.5 µg/mL, 5 µg/mL, 10 µg/mL or 20 µg/mL after 30 minutes of incubation did not influence the degradation profile when tested with 0.7 nM t-PA straight away (data not shown), but it did improve the thrombolysis with 0.7 nM t-PA after 3 h and 30 minutes additional incubation. The degradation profiles on Fig. 5A show that the addition of Reopro at the beginning of the maturation of the clots reduces, in a dose dependent manner, the thrombolysis resistance conferred by maturation time. With 20 µg/mL Reopro this achieved a degradation profile close to the one obtained with the same t-PA dose on fresh clots (shown in dotted line). The activation times and the clot lysis rates of aged clots were influenced by the addition of Reopro at the beginning of the maturation, as presented in Fig. 5B and C. With the addition of 20 µg/mL Reopro, the activation time from 0.7 nM tPA on aged clots (37 ± 11 min) was similar to the activation time obtained on fresh clots (40 ± 16 min). The addition of 20 µg/mL Reopro was further tested on the thrombolysis of aged clots by plasmin and urokinase. With urokinase, the inhibition of GPIIb/IIIa restored the activation time for aged clots to the level of the fresh clots (Fig. 5E and F, 12 ± 6 min with 200 U/mL urokinase on fresh clots and 12 ± 4 min with 200 U/mL urokinase on aged clots supplemented with 20 µg/mL Reopro). With plasmin, the addition of Reopro did not influence the effect of maturation on the thrombolysis profile (Fig. 5G).

## Discussion

This study reports the implementation and the validation of a new method to measure the extent and the rate of clot lysis obtained from thrombolytic drugs of choice.

We validated the technique by measuring the thrombolysis obtained from the addition of different doses of plasmin, urokinase and tissue plasminogen activator (t-PA) on halo clots incubated for 30 minutes at 37 °C. The three enzymes provided precise and reproducible degradation curves. The thrombolysis profiles obtained with plasmin have short activation times, which reflects the direct action of plasmin on the fibrin network. We attribute the observed saturation effect to the presence of plasmin inhibitors in the blood (α2-macroglobulin and α2-antiplasmin)^27, 28^. The plasminogen activator (PA) samples exhibited a different lysis profile, corresponding to a two-step process; the PA converts the endogenous plasminogen into plasmin, which in turn degrades the fibrin network. Accordingly, the higher the dose of PA, the faster that plasmin was generated, and the shorter the activation time was. These results demonstrate that the assay enables precise assessment of the kinetic action of thrombolytic drugs. Our group used the assay in recent studies to determine the thrombolytic activity of thrombin degradable capsules and thrombin activatable microplasminogen^29, 30^. The thrombolysis obtained from exogenous thrombolytic drugs in this study was similar to that obtained with different traditional methods of studying fibrinolysis. Genét *et al*. measured 80.19 ± 3.79% thrombolysis at 1 hour using 1.8 nM t-PA with kaolin thromboelastography (TEG), 79.88 ± 9.35% with Rapid TEG, and 91.55 ± 4.09% with Functional fibrinogen TEG. With our assay we measured at 1 hour after addition of 1.4 nM t-PA a thrombolysis of 81.83 ± 4.04%^31^. In another recent trial to assess the capacity of TEG to detect hyperfibrinolysis, thrombolysis at 30 minutes of 8.2% was obtained after the addition of 75 ng/mL t-PA with blood from a healthy cohort, and we measured with our assay a thrombolysis of 3.63 ± 14.56% at 30 minutes after the addition of 1.4 nM t-PA (i.e. 96 ng/mL)^32^. With the plate turbidometry method from plasma clot lysis, Xin *et al*. measured a time to 50% lysis of around 60 minutes with a t-PA dose of 0.03 µg/mL, and we obtained a T0.5 value of 57.6 ± 13.8 minutes with a t-PA dose of 0.7 nM (i.e. 48 ng/mL)^33^.

Maturation time of halo clots had a pronounced impact on the thrombolysis profiles. With urokinase, activation time was significantly delayed and maximal clot lysis rate was significantly reduced on aged clots. This is in accordance with the resistance to lysis from plasminogen activators conferred by clot maturation that is reported in other studies^25, 26^. Interestingly, the activation time was increased following a shorter maturation time (2 h), compared to the maximal clot lysis rate, which was only affected following the longer maturation time (4 h). This observation might indicate that these 2 parameters are affected by different aspects of clot maturation. The activation time would be more affected by platelet contraction, which reduces the porosity of the blood clot and thereby the penetration of the plasminogen activators. The maximum clot lysis rate would be more dependent on the perturbation of the proteins involved in the fibrinolytic system as a result of the maturation process. With t-PA, clot maturation did also affect the thrombolysis profile, but in a less pronounced manner. Although urokinase and t-PA both act by cleaving plasminogen to generate plasmin, they exhibit some differences in kinetics and fibrin dependency, which might be responsible for the difference we observe here^34^. With plasmin, the degradation was not slowed down by clot maturation. It was even accelerated at 0.1 and 0.5 U/mL, and the maximal degradation went from 80 ± 4% on fresh clots to 95 ± 3% (*p < 0.05) on aged clots. It seems that in our model it is mainly factors affecting plasminogen activation that differ between fresh clots (30 min) and old clots (4 h). Whether this is explained by a different extent of platelet contraction or secretion, by a modification of fibrin structure and availability, by a different general clot stiffness and/or a variation of activity, or by stability, the degree of activation of the proteases involved at different stages of haemostasis remains unknown at this stage. However, the blockage of the platelet GPIIb/IIIa receptor at the beginning of the maturation process seems to prevent the thrombolysis resistance conferred by the maturation. The GPIIb/IIIa complex is involved in platelet aggregation and GPIIb/IIIa blockers have been reported to reduce clot contraction^35^. Thus, the tendency of Reopro to reduce the clot lysis resistance of aged clots in the assay could be explained by a reduction in platelet and clot contraction. This hypothesis would explain why the lysis from exogenous plasminogen activators is hindered by maturation, whereas the lysis from plasmin is not. Indeed, the higher contraction resulting from longer maturation would logically be associated with lower penetration of the enzymes added exogenously. This would not affect degradation from plasmin as it has a direct fibrinolytic activity, but plasminogen activators need to penetrate the clot to cleave the endogenous plasminogen into active plasmin.

It should be noted, however, that the halo assay model enables maturation of clots in static conditions and would therefore not be affected by mechanisms involving flow. Muthard *et al*. reported evidence that the hemodynamic state of blood flow plays a role in thrombus contraction and permeability^36^. Moreover, the crosslinking of α2-antiplasmin to fibrin via the activated plasma transglutaminase factor XIII, reported to be the principal mechanism whereby plasmin-mediated clot degradation is minimised^37^, could have no effect at all in our static model, as the amount of α2-antiplasmin at the clot area would simply correspond to the amount present in the 25 μL of blood added into the well^38, 39^. The activity of this fixed amount of α2-antiplasmin could potentially be compromised after 4 hours incubation, explaining the higher degradative activity of plasmin on aged clots than on fresh clots. All things considered, the halo assay seems to reproduce some aspects of maturation that affect thrombolysis from plasminogen activators. However, one should be cautious drawing any conclusions on the pathways involved in the formation, the maturation, and the degradation of the halo clots. Compared to other techniques our assay allows very precise maturation, and we underline the importance of incubation time to perform it properly. Our model has limitations due to its static nature and the need to supplement with calcium chloride and tissue factor with phospholipids, which generates large amounts of thrombin. Investigators might consider forming the clots via different pathways if required.

On the other hand, this novel technique offers many advantages over the current methods available to measure thrombolysis in static conditions. With the improvements provided by the free oscillation rheology (FOR), methods based on clot viscoelasticity now enable precise measurements of clot lysis time, which might improve diagnosis of hyperfibrinolysis in a clinical setting^10, 40^. However, some concerns about the low repeatability of the FOR method were also reported^41, 42^, and a different approach to measure thrombolysis in whole blood samples should then still be considered. The halo assay also enables high throughput measurement (96 samples instead of 4) using 25 µL of whole blood per sample, compared to 300 µL for viscoelastic based methods, which would offer clinicians an important increase in replicates and conditions tested.

The main current alternative to viscoelastic methods, often preferred for the high throughput screening they provide, are the euglobulin clot lysis time (ECLT) assays. Their obvious limitation is the exclusion of all blood cells from the clots, and this assay is therefore limited to the study of fibrinolysis rather than thrombolysis. It should also be noted that the euglobulin fraction isolated from whole blood samples contains a very different protein composition with respect to the fibrinolytic system; Smith *et al*. reported that the euglobulin fractions of their study recovered 71% of the initial fibrinogen, 50.3% of the FVIII, 38.5% of the TAFI, 42.4% of the PAI-1, 90.8% of the tPA, 65.2% of the plasminogen and only 7.1% of the α2-antiplasmin^13^. The halo assay offers the same large screening possibilities, but with lysis profiles more relevant to the physiopathology of thrombosis, as the clots are obtained from whole blood samples. In addition, although some optimization of the method has reduced the time of the protocol, ECLT assays measuring fibrinolysis still require several hours^13, 43, 44^, whereas the method we propose can provide an answer within one hour (including the clot formation time), which is more suitable for the clinical setting.

An important interest of *in vitro* thrombolysis assays is to improve clinical care through the rapid assessment of haemostatic defects. The thromboelastography and the rotational thromboelastometry techniques have been proposed to identify hyperfibrinolysis after trauma and guide therapy, but the outcomes are not conclusive^45, 46^. The precise analysis of thrombolysis rate provided by the method presented in this paper could effectively upgrade this guidance therapy approach. It could potentially help to estimate altered fibrinolysis in several other pertinent clinical situations such as liver or bone marrow transplantation, liver cirrhosis, and haemophilia, or to assess the haemorrhagic risk after thrombolytic therapy^47–50^. The assay has an intra-assay coefficient of variation of 5.15% which is similar to that reported for the thromboelastrography methods^41, 51^ (3 to 6%), but higher than that for the optimised ECLT assay^13, 44^ (0.7 to 2.6%). Although the halo assay in its current form has encouraging reproducibility, we believe the standardization could be further improved by using a specific microtiter plate with wells designed to form the halo thrombi by fluidic cohesion, together with an automated liquid handling system. Fluctuations in the assay outcome may also result from the broad concentration variation of blood components involved in thrombolysis between donors. Adjustment of the thrombolysis profiles from measurement of those components might also contribute to improving the precision of the method further.

Further studies will be addressing the standardisation requirement issue, and assessing the feasibility of identifying fibrinolysis disturbance in patient blood samples with this technique, paving the way for adaptation as a point-of-care test.

## Conclusion

The method described constitutes a novel, broadly accessible, and useful tool for thrombosis research. Precise and reproducible thrombolysis profiles were measured from various concentrations of plasmin and plasminogen activators (urokinase and tissue type) added onto thrombi obtained from human whole blood. This novel implementation is well adapted to measure the influence of thrombus maturation and key parameters such as activation time and thrombolysis rate. It also overcomes several limitations of the current traditional thrombolysis/fibrinolysis techniques by reducing drastically the blood volume required and by providing high throughput screening on a whole blood based assay. In addition, the rapid, high throughput and accurate measurement of thrombolysis provided by the technique could potentially be adapted to the clinic as a point-of-care assay to improve the diagnosis of thrombolysis disorders.

## Methods

### Halo thrombus formation

The recruitment of participants and collection of blood specimens was approved by the Alfred Hospital Ethics Committee (Project 67/15), ensuring that human blood collection and research were conducted in accordance with Australian regulations. Human blood was collected with low level of risk from healthy volunteers who had not taken medications which may affect haemostasis in the past 10 days via venepuncture into tri-sodium citrate (3.2% w/v final). Signed informed consent was obtained prior to participation. Clot formation was induced with recombinant tissue factor, supplemented with synthetic phospholipids (Dade^®^ Innovin^®^, Siemens, Munich, Germany). Innovin vials were reconstituted according to manufacturer’s instructions into deionised water, and a calcium concentration of 30 mM was determined by complexometric titration with Eriochrome Black T (EBT, Sigma) and EDTA. A clotting mixture was freshly prepared from Innovin (15% v/v) and calcium chloride (to a final calcium concentration of 67 mM) in PBS, and 5 μL of this mixture was deposited onto the bottom edge of the wells of a flat-bottomed, transparent, nonpyrogenic, polystyrene, tissue culture treated, sterile, 96-well plate (Costar, FisherScientific, Pittsburgh, Pennsylvania, US) (Fig. 1A). It should be noted that the combination of phosphate buffer with calcium ions can result in calcium-phosphate precipitate formation, and the PBS may here be replaced by HEPES buffer (25 mM HEPES, 137 mM NaCl) to avoid this potential issue. Blood clots were formed around the edge of the bottom of the wells by the addition of 25 μL of blood with a P100 micropipette (Eppendorf, Life Sciences Biotechnology, Hamburg, Germany). To obtain a homogenous blood clot the 5 μL of the clotting mixture must be spread in a circular motion around the edges of the well with the extremity of the tip containing the blood (Fig. 1B) just before releasing slowly the 25 μL of blood. This will then follow the same circular motion along the edge thanks to fluidic cohesion effect (Fig. 1C). The clots should have a homogenous halo shape at the bottom of the wells, leaving the centre area of the well empty, as shown in Fig. 1D. The plate was sealed and incubated in a 37 °C incubator for 30 minutes (fresh clots), 1, 2, 3 or 4 hours (aged clots).

### Spectrophotometric reading of fibrinolysis

Fibrinolysis rates were tested using different activities of human plasmin (0.001 U, 0.01 U and 0.05 U, Sigma,St. Louis, Missouri, US), urokinase from human urine (10, 20 and 40 U, Sigma) and recombinant human tissue plasminogen activator (t-PA, 0.035 pmol, 0.07 pmol, 0.14 pmol, 0.35 pmol, 0.7 pmol, Boehringer Ingelheim GmbH, Rhein, Germany) diluted into 70 μL of PBS. The 70 μL samples were added simultaneously into the wells containing the halo clots with a multichannel pipette (Xplorer^®^ plus, Eppendorf, Life Sciences Biotechnology, Hamburg, Germany). The total final volume is 100 μL in each well, so the final concentrations are therefore 0.01 U/mL, 0.1 U/mL and 0.5 U/mL for plasmin, 100 U/mL, 200 U/mL and 400 U/mL for urokinase and 0.35 nM, 0.7 nM, 1.4 nM, 3.5 nM and 7 nM for t-PA. Straight after the addition of samples the degradation of the halo clots was measured with a plate reader (EnSpire Multimode, PerkiElmer, Waltham, Massachussetts, US) via the absorbance change at 510 nm caused by blood progressively covering the centre of the well (Fig. 1E). The fibrinolysis rate was assessed by one measurement at 510 nm every minute, with 5 seconds orbital shaking (200 rpm, 3 mm diameter) at each time point, over 2 hours at 37 °C. Negative controls for the assay were obtained from the addition of 70 μL of PBS to halo thrombi (no fibrinolytic drug), and positive controls consisted of a well containing 25 μL of blood and 75 μL of PBS (no clotting mixture). The positive control wells provided absorbance values corresponding to full degradation (A_total_), and the negative control wells provided reading for no degradation (A_zero_). At each time point, the percentage of degradation were obtained from this formula: D_x_(t) = 100(A_x_(t) − A_zero_(t))/(A_total_(t) − A_zero_(t)). Replicates were obtained with clots made from the blood of 5 different donors, incubated for 30 minutes (fresh clots) or 4 hours (aged clots), and mean values ± SEM were plotted over time. SEM is used for the measurement of the estimated mean thrombolysis profile. To study the role of GPIIb/IIIa, different doses of Abciximab (Reopro, Jansen Biologics BV, Leiden, Netherlands) were added on top of the halo clots at 30 minutes incubation and thrombolysis was measured straight away, or at 4 hours incubation with t-PA at 0.7 nM, urokinase at 200 U/mL or plasmin at 0.1 U/mL.

### Analysis of the fibrinolysis profiles

Specific parameters were established to support the reading of the fibrinolysis profiles obtained with the halo assay. The maximum degradation (D_max_) is the maximum value of degradation obtained over the experiment. The maximum clot lysis rate (CLR_max_) reached over the experiment corresponds to the maximum value of D_x_(t)/dt and was determined on the first derivative analysis of the degradation profile. The activation time (A_t_) required to induce lysis was defined as the first value of t (in minutes) for which D_x_(t)/dt > 1. T0.5 (time elapsed until 50% lysis) was obtained from the intersection of dotted line and lysis curves. A_t_, CLR_max_ and T0.5 values were measured for urokinase, t-PA and plasmin samples (no A_t_ for plasmin) on clots incubated for 30 min or 4 h at each concentration and in each replicate, and are presented as mean values ± SD (n = 5). SD is used to depict the variability of the different parameters measured between individuals.

### Statistical analysis

All statistical analysis was performed with GraphPad Prism V6 (GraphPad Software, San Diego, California, US). Intraindividual variation coefficients were calculated with the formula CV = SD/mean from experiments where the same doses of plasmin and urokinase were tested in triplicate. The mean intraindividual variation coefficient of the method was measured from the mean value of the variation coefficients obtained from 3 independent experiments. The interindividual variation coefficients were calculated from the SD and mean values obtained from the same doses of plasmin and urokinase on 5 different donors. CLR_max_, A_t_, and T0.5 mean values from different doses of urokinase, plasmin or t-PA obtained with 30 minutes incubated clots were compared with repeated-measures one way ANOVA and Turkey’s multiple comparison tests. T0.5 mean values from different doses of plasmin obtained with 30 minutes incubated clots were compared with a paired t test. A_t_, T0.5 and CLR_max_ mean values from thrombolysis profiles of aged clots were compared to A_t_, T0.5 and CLR_max_ mean values obtained from fresh thrombi with repeated-measures two-way ANOVA and Sidak’s multiple comparisons test. For the degradation of fresh clots versus aged clots the mean time to reach full degradation with 0.5 U/mL plasmin and the mean D_max_ value with 0.1 U/mL plasmin was compared with a paired t test. A difference of p < 0.05 was considered significant.
